# CPStools: A package for analyzing chloroplast genome sequences

**DOI:** 10.1002/imo2.25

**Published:** 2024-08-23

**Authors:** Lijin Huang, Huanxi Yu, Zhi Wang, Wenbo Xu

**Affiliations:** ^1^ School of Traditional Chinese Pharmacy China Pharmaceutical University Nanjing China; ^2^ Nanjing Institute of Environmental Sciences Ministry of Ecology and Environment (MEE) Nanjing China

## Abstract

CPStools is a user‐friendly software for comprehensive chloroplast genome analysis. It integrates 10 functionalities including Genbank file checking, statistical information generation, sequence adjustment, inverted repeat (IR) regions identification, nucleotide diversity (Pi) analysis, relative synonymous codon usage (RSCU) calculation, simple sequence repeats (SSRs) identification, long sequence repeats (LSRs) statistics, phylogenetic analysis, and format conversion. CPStools handles Genbank or Fasta format inputs, delivering results comparable to other tools while excelling in data preparation for advanced analysis. It uniquely generates consensus merged protein‐coding sequence (CDS) or protein sequences from multiple Genbank files, facilitating advanced phylogenetic analysis. CPStools offer reliable results for comprehensive chloroplast genome analysis.

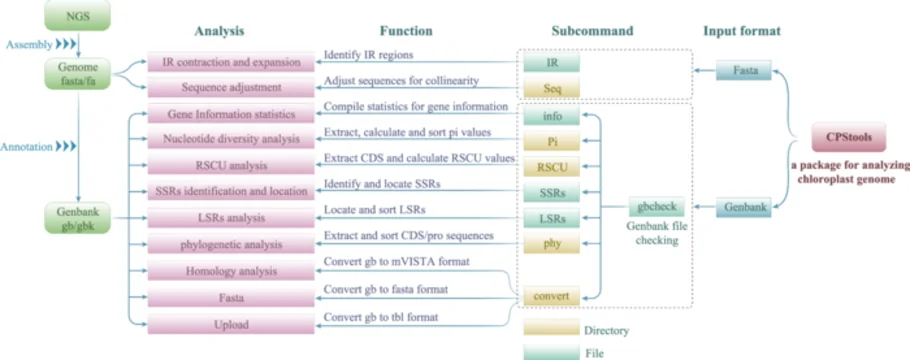

Chloroplasts are essential organelles in green plants and algae for photosynthesis [1]. Chloroplast genomes are typically circular, and are featured by a quadripartite structure with a small single‐copy (SSC) region, a large single‐copy (LSC) region, and two inverted repeat (IR) regions [2]. These genomes are pivotal in phylogenetic classification and species identification. With advancements in next‐generation sequencing technology, chloroplast genome analysis has become routine. However, current tools for chloroplast genome analysis have notable limitations. For instance, MIcroSAtellite Identification (MISA) is widely used for detecting simple sequence repeats (SSRs), but it involves complex categorization that can be challenging for inexperienced users [3]. CodonW, used for calculating relative synonymous codon usage (RSCU) values, requires a time‐consuming process to prepare the necessary inputs, such as extracting consensus protein‐coding sequences (CDS) and filtering short sequences from multiple Genbank files [4]. Additionally, Geseq and Geneious, when used for identifying chloroplast genome regions, often produce inaccurate results due to short IR fragments [5, 6]. There is a clear need for efficient tools to provide accurate results and prepare data for advanced analysis, such as nucleotide diversity (Pi) and phylogenetic analyses.

To address these challenges, we developed CPStools, which integrates 10 subcommands, and each one offers specific functionalities, overcoming the limitations of existing tools. By simplifying input requirements and automating complex processes, CPStools significantly enhances the efficiency and accuracy of chloroplast genome analyses. This streamlined approach not only saves considerable time for researchers but also reduces the likelihood of errors, making CPStools an important contribution in chloroplast genome studies.

## 1 RESULTS

**Figure 1:**
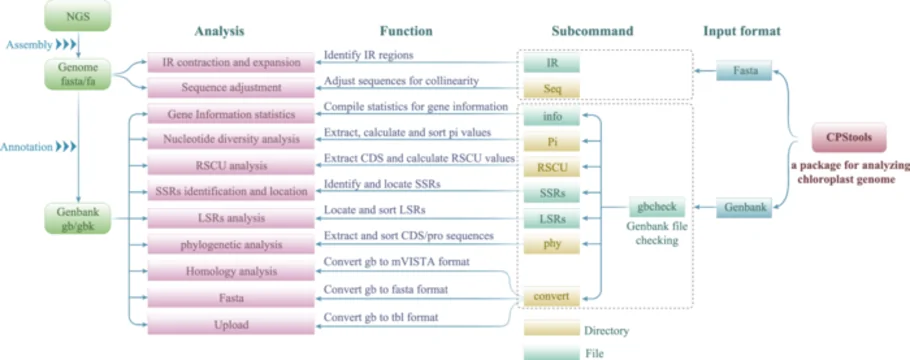
The workflow of CPStools. The analysis represents common chloroplast genome analyses. Subcommand and Function are CPStools subcommands and their corresponding functions. Cyan subcommands accept single files as input, while yellow subcommands accept directories.

**Figure 2:**
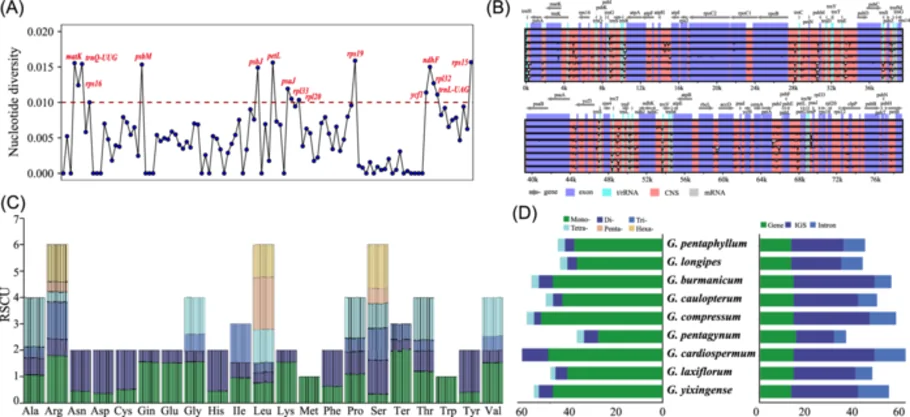
The results generated from CPStools. (A) Nucleotide diversity (Pi) analysis of genes. (B) Partial conversion and visualization of mVISTA. (C) Relative synonymous codon usage (RSCU) analysis. (D) The identification and location of simple sequence repeats (SSRs).

CPStools addresses 10 core functionalities which are essential for comparative genomic studies (Figure 1). These functions include Genbank file checking, statistical information generation, sequence adjustment, IR regions identification, Pi analysis, RSCU calculation, SSRs identification, long sequence repeats (LSRs) statistics, phylogenetic analysis, and format conversion. Nine sequences downloaded from NCBI were analyzed using CPStools for comparative analysis. During the analysis, 13 genes were identified that do not start with “ATG.” All nine chloroplast genomes were annotated with 113 unique genes, except for *Gynostemma yixingense*, which had an incorrect annotation in the *trnf*M‐CAU and *trn*M‐CAU genes, a common error among inexperienced researchers. The “IR” subcommand was used for boundary detection, revealing that two of the nine sequences do not start with the first base pairs in the LSC region. In Geseq and Geneious, the nine sequences all start with the first base pairs in the LSC region, however, the short repeats cannot be identified accurately (Table S1). Combining the co‐linear results and IR identification results, the “Seq” subcommand easily adjusted the sequences. Pi analysis was performed with “Pi” subcommand, extracting 110 shared single genes and 150 intergenic regions. After multiple alignments and calculating the pi values, regions with high pi values were selected as barcode regions for identification purposes (Figure 2A). The conversion from GenBank to mVISTA input was also accurately visualized using mVISTA (Figure 2B). Except for 51 genes in *G. yixingense*, the other eight species all had 52 genes retained, and the RSCU values were calculated with the RSCU subcommand (Figure 2C). Then, 44, 55, 52, 58, 37, 62, 47, 54, and 45 SSRs were identified in the nine *Gynostemma* species. The locations of these SSRs in the IGS, intron, and exon regions were detected, along using the analysis of LSRs (Figure 2D, Tables S2 and S3). All analyses were completed in half an hour with high accuracy.

## 2 DISCUSSION

CPStools represents a breakthrough in chloroplast genome analysis, providing a user‐friendly platform for rapid and comprehensive analysis with reliable results. It surpasses tools like Geseq and Geneious in identifying tetrad structures and handling short sequences, providing rapid results. Unlike labor‐intensive processes required for DNAsp6 and CodonW, CPStools can efficiently extract shared gene sequences and batch‐adjusting sequences by simplifying file preparation and supporting batch pi calculations. This significantly reduces research workflow time and makes CPStools highly advantageous for researchers. Continuous refinement and feature expansion are planned.

CPStools relies on Biopython for parsing GenBank and Fasta files, which must strictly adhere to standard format specifications [7]. We recommend using CPGAVAS2 for annotation, as results from other software may not match CPStools due to format discrepancies [8]. The subcommand “gbcheck” checks GenBank files and adjusts them to the standard format required by CPStools. Researchers should ensure data compatibility and accuracy when using CPStools.

## 3 CONCLUSIONS

CPStools represents a significant advance in the field of chloroplast genome analysis by integrating 10 essential functionalities into a single, user‐friendly package. This tool simplifies and automates complex processes, significantly enhancing the efficiency and accuracy of chloroplast genome studies. By addressing the limitations of existing tools, CPStools provides reliable and comprehensive results, facilitating detailed genomic analyses and phylogenetic studies. The incorporation of features such as sequence adjustment, nucleotide diversity analysis, codon usage calculation, and repeat identification ensures that researchers can conduct thorough research in the least amount of time. Future developments will continue to expand its capabilities, making CPStools an important resource for researchers in the field of chloroplast genomics.

## 4 METHODS

### Genbank files check and statistics

The “gbcheck” subcommand offers two modes: self‐checking Genbank files and comparative analysis with reference file. In the self‐checking mode, the script examines CDS genes, assesses start and stop codons, and identifies multiple stop codons. Comparative mode compares annotation files through identifying discrepancies in gene annotation. The “info” subcommand provides statistical analysis of gene counts, types, and exon numbers, which is crucial for detailed genomic element statistics, speeding up and improving the accuracy of chloroplast genome annotation.

### Sequence adjustment

The chloroplast genome's circular topology allows segmentation at arbitrary locations to yield linear sequences. Challenges arise when the IR region is split into fragments with only a few base pairs. The “IR” subcommand, using a seed size of 1000 base pairs, ensures accurate identification of the four chloroplast genome regions. The “Seq” subcommand provides modes for sequence adjustments: LSC aligns to the first base pair in the LSC region start, SSC orients the SSC region forward, and RP implements reverse complementation, positioning the first base pair in LSC at the sequence outset.

### Pi analysis

The pi analysis detects polymorphisms within sequences, with regions of high mutation rates serving as genetic markers for species differentiation. Extracting sequences from gene and intergenic spacer (IGS) regions is challenging, and computing pi values via DNAsp6 is time‐consuming because it only accepts a single multiple sequence alignment file for calculation [9]. This process is further complicated by the presence of over 200 consensus sequences extracted from the entire chloroplast genome. The “Pi“ subcommands streamline this analysis by identifying and extracting consensus sequences from gene and IGS regions, supporting batch pi value computation, and organizing results by their location within chloroplast genomes.

### RSCU, SSRs, and LSRs analyses

RSCU analysis, essential for understanding codon bias in chloroplast genomes, traditionally involves time‐consuming steps, including filtering the lengths of conserved protein‐coding sequences, excluding repetitive sequences, and computing relative codon usage frequencies. The “RSCU” subcommand allows rapid and accurate RSCU value calculation from multiple Genbank files.

The “SSRs” subcommand accurately identifies SSRs using preset minimum lengths for different types: 10 for mononucleotides, 6 for dinucleotides, 5 for trinucleotides, and 4 for tetranucleotides, pentanucleotides, and hexanucleotides. It also locates each SSR within gene, intron, or IGS. The “LSRs” subcommand pinpoints each LSR within genomic structures, offering a clearer understanding of genomic variations.

### Phylogenetic analysis

Phylogenetic analysis is primarily based on three types of data: the entire chloroplast genome, consensus CDS, and protein sequences. The “Seq” subcommand efficiently obtains and merges the complete chloroplast genome sequence. The “phy” subcommand facilitates the extraction and combination of shared CDS and protein sequences, preparing them for phylogenetic analysis. These sequences, following multiple alignments, are prepared for phylogenetic analysis.

### Format conversion

CPStools supports the conversion of gb files into tbl, Fasta, and mVISTA annotation formats. The “convert” subcommand supports these conversions, with tbl format for NCBI database uploads and mVISTA format for mVISTA software input [10].

## AUTHOR CONTRIBUTIONS

**Lijin Huang**: Conceptualization; software; data curation; visualization; validation; writing—original draft; formal analysis. **Huanxi Yu**: Conceptualization; methodology; funding acquisition; investigation; data curation; writing—review and editing. **Zhi Wang**: Conceptualization; methodology; funding acquisition; visualization. **Wenbo Xu**: Conceptualization; investigation; writing—original draft; writing—review and editing; visualization; validation; methodology; software; formal analysis; project administration; data curation; supervision; resources.

## CONFLICT OF INTEREST STATEMENT

The authors declare no conflict of interest.

## ETHICS STATEMENT

No animals or humans were involved in this study.

## Supporting information


**Table S1:** Comparison of chloroplast genome tetrad structure identification results using CPStools, Geseq, and Geneious.
**Table S2:** Comparison of SSRs identified results from CPStools and MISA website.
**Table S3:** Comparison of LSRs identified results from CPStools and Reputer website.

## Data Availability

CPStools and its dependencies are coded in Python, and the source code is available at GitHub (https://github.com/Xwb7533/CPStools). Sample data for each function is provided in the test data directory, along with a detailed help documentation. Additionally, video tutorials on how to use CPStools can be found on Bilibili (https://www.bilibili.com/video/BV1fZ421K7nw). Supplementary materials (tables, graphical abstract, slides, videos, Chinese translated version, and update materials) may be found in the online DOI or iMeta Science http://www.imeta.science/imetaomics/.
